# Intraoperative angiography should be standard in cerebral aneurysm surgery

**DOI:** 10.1186/1471-2482-9-7

**Published:** 2009-04-30

**Authors:** Jonathan A Friedman, Ravi Kumar

**Affiliations:** 1 Texas A&M Health Science Center College of Medicine, 3201 University Drive, Suite 425, Bryan, TX 77802 USA

## Abstract

Intraoperative angiography (IOA) has proven to be a safe and effective adjunct to surgical repair of cerebral aneurysms. Substantial practice variation exists regarding use of this modality in different centers, including use of IOA routinely, selectively, or rarely. In this editorial, we discuss our experience and review the existing literature to develop an argument for routine use of IOA during cerebral aneurysm surgery.

## Editorial

Intraoperative angiography (IOA) has been considered since the 1960s for use during surgical repair of cerebral aneurysms. The potential to confirm complete aneurysm occlusion and patency of the parent vasculature intraoperatively are the key clinical uses that underlie IOA. However, no consensus exists regarding routine use of IOA in cerebral aneurysm surgery. Different institutional paradigms include use of IOA rarely or never, selectively based on aneurysm complexity, or routinely in all aneurysm cases.

**Figure 1:**
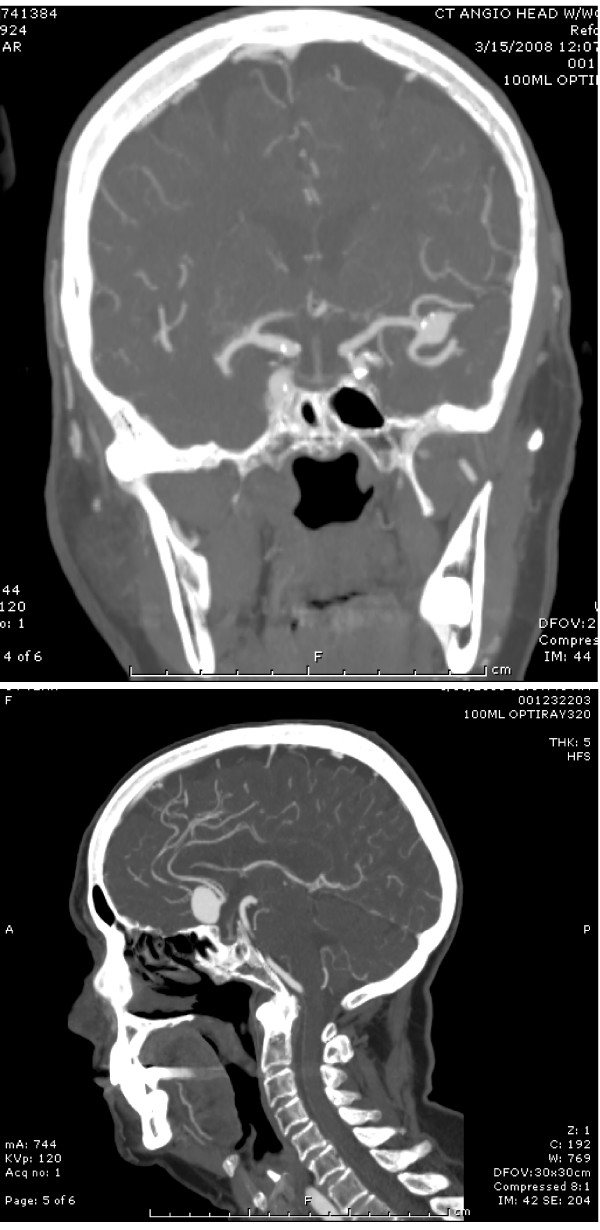
**Anteroposterior (a) and lateral (b) views of CT angiogram**. These images demonstrate large middle cerebral artery aneurysm and giant anterior communicating artery aneurysm.

**Figure 2:**
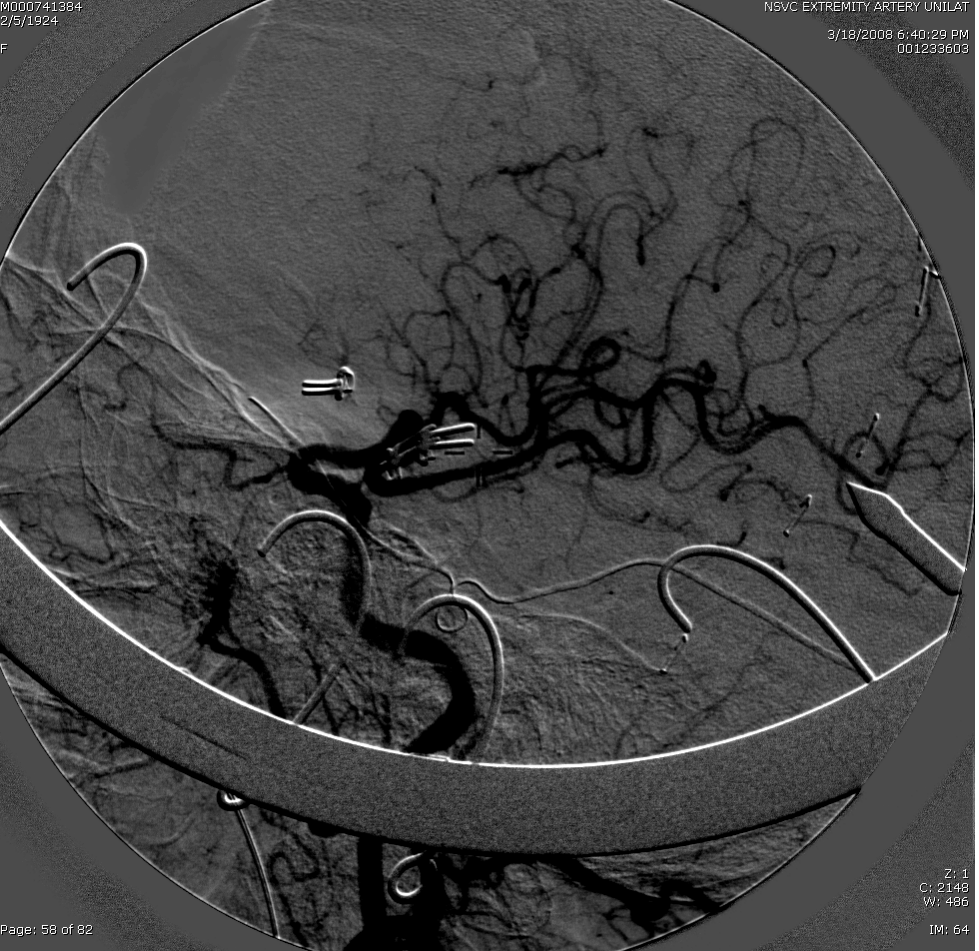
**Intraoperative angiogram demonstrating occlusion of middle cerebral artery aneurysm with patency of middle cerebral branches**. The anterior communicating artery aneurysm is also occluded, but there is no filling of the anterior cerebral artery, suggesting parent vessel occlusion by the clip.

**Figure 3:**
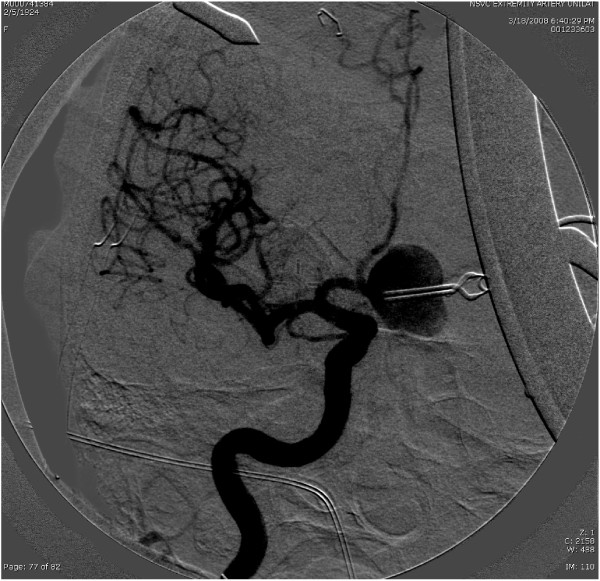
**Intraoperative angiogram after clip revision**. Flow in the parent vessel has been restored, but the aneurysm now fills.

The fundamental value of IOA during aneurysm repair is to confirm complete occlusion of the aneurysm, and to demonstrate patency of surrounding vasculature (Figure 1, 2 and 3). As many as 5% – 7.3% of surgically treated aneurysms are unexpectedly incompletely occluded, leading to additional treatment or to ongoing risk of rupture.[1,2] Without IOA, the surgeon's only means to confirm complete aneurysm occlusion is to puncture the aneurysm dome – an incompletely occluded aneurysm will hemorrhage from the puncture site, which although not usually problematic is certainly not an optimal means to discover incomplete aneurysm occlusion. Parent vessel occlusion during surgical clipping of a cerebral aneurysm occurs in approximately 3 – 9% of cases.[1,3-5] Although rare, parent vessel occlusion can lead to permanent neurological deficit or death. Furthermore, only a small window of time exists in which to recognize parent vessel occlusion and restore flow before irreversible ischemia of brain parenchyma will occur, such that postoperative investigations demonstrating parent vessel occlusion do not generally lead to successful intervention. Because IOA can demonstrate both incomplete aneurysm occlusion and parent vessel occlusion in a facile and timely manner, and lead to corrective intervention during the initial craniotomy, IOA has significant potential to improve surgical outcomes.

Intraoperative angiography is both effective and accurate. In a single-center study of 517 aneurysms that underwent IOA during cerebral aneurysm surgery, a 12.4% revision of clip placement due to abnormalities found on IOA was reported.[3] The largest percentage of parent vessel occlusions were related to aneurysms of the proximal internal carotid artery. Aneurysms greater than 15 mm were independent predictors for the need for clip revision. The same study looked at the accuracy of IOA: the accuracy rate of IOA in detecting abnormalities as compared to postoperative angiography was 95%.[3]

While surgical repair of large and/or complex aneurysms carries a higher likelihood of findings on IOA leading to clip revision, even experienced surgeons cannot adequately predict the need for IOA on a selective basis. In a study of 200 patients treated at an experienced center, all of whom underwent IOA, 20% were predicted by the surgeon to need IOA, while 80% were predicted to not need IOA. The prediction was based on location, size, and unique features.[6] Of the 159 cases not predicted to need IOA, 7 (4.4%) needed clip revision due to aneurysm remnants, parent vessel occlusions, and undiagnosed aneurysms. Even in the most experienced hands, the potential for abnormal findings on IOA cannot be predicted with enough accuracy to justify selective use.

The potential value of IOA must be weighed against the morbidity and practical challenges of the procedure. Complications of IOA include stroke, dissection, emboli, arterial dissection, and hematoma. Complication rates have been reported in the range of 0.4% to 2.6%, and in our experience the risk of IOA was 3%. [2-5] While this represents increased morbidity compared to conventional cerebral angiography, these complication rates compare favorably to the rate of complications of surgical aneurysm repair which may be amenable to correction with IOA.

The biggest practical challenge of performing IOA routinely is having a skilled practitioner available to perform the procedure. This is particularly the case for night and weekend surgery. In our experience with implementing IOA routinely at two institutions, lack of radiology support led to inability to perform IOA in 35% of cases. [4] For this reason, implementation of a program of routine IOA requires a small number of "champions" that are committed to performing the procedure irrespective of schedule. The speed with which the IOA can be obtained is also a critical practical consideration, since in the event of parent vessel occlusion rapid restoration of flow is required to prevent stroke. We generally place a sheath in the femoral artery in the angiography suite before the operation, to facilitate rapid vascular catheterization and angiography intraoperatively. The responsiveness and coordination of the radiology team is also critical in the timeliness of results. This coordination is facilitated by performing IOA routinely, to build the experience and refine the systems. Other causes for not obtaining IOA in our experience included lack of radiolucent head holders, positioning of the patient in the lateral position, trapping or wrapping of the aneurysm, and individual patient contraindications to angiography. [4]

It is our opinion that IOA should be performed routinely on all patients undergoing cerebral aneurysm surgery. Morbidity due to incomplete aneurysm occlusion and/or parent vessel occlusion is not infrequent and is clinically important, and both complications may be avoided in certain cases by IOA. The limited morbidity of IOA is greatly outweighed by these potential benefits. Performing IOA in selective cases will not maximize the potential value since the need for IOA cannot be completely predicted.[5] Even the simplest aneurysm repair in the most experienced hands can have complications potentially amenable to discovery by IOA with consequent correctional intervention. Finally, optimization of reliability, speed, and efficacy of IOA can only be achieved within an institution when its use is routine.

## Pre-publication history

The pre-publication history for this paper can be accessed here:


